# Capsules of virulent pneumococcal serotypes enhance formation of neutrophil extracellular traps during *in vivo* pathogenesis of pneumonia

**DOI:** 10.18632/oncotarget.8451

**Published:** 2016-03-28

**Authors:** Anandi Narayana Moorthy, Prashant Rai, Huipeng Jiao, Shi Wang, Kong Bing Tan, Liang Qin, Hiroshi Watanabe, Yongliang Zhang, Narasaraju Teluguakula, Vincent Tak Kwong Chow

**Affiliations:** ^1^ Department of Microbiology and Immunology, Yong Loo Lin School of Medicine, National University of Singapore, Kent Ridge, Singapore; ^2^ Infectious Diseases Interdisciplinary Research Group, Singapore-Massachusetts Institute of Technology Alliance in Research and Technology, Singapore; ^3^ Department of Pathology, National University Hospital, Singapore; ^4^ Department of Infection Control and Prevention, Kurume University School of Medicine, Fukuoka, Japan; ^5^ Center for Veterinary Health Sciences, Oklahoma State University, Stillwater, OK, USA

**Keywords:** neutrophil extracellular traps, Streptococcus pneumoniae, capsule, serotype, secondary pneumonia, Immunology and Microbiology Section, Immune response, Immunity

## Abstract

Neutrophil extracellular traps (NETs) are released by activated neutrophils to ensnare and kill microorganisms. NETs have been implicated in tissue injury since they carry cytotoxic components of the activated neutrophils. We have previously demonstrated the generation of NETs in infected murine lungs during both primary pneumococcal pneumonia and secondary pneumococcal pneumonia after primary influenza. In this study, we assessed the correlation of pneumococcal capsule size with pulmonary NETs formation and disease severity. We compared NETs formation in the lungs of mice infected with three pneumococcal strains of varying virulence namely serotypes 3, 4 and 19F, as well as a capsule-deficient mutant of serotype 4. In primary pneumonia, NETs generation was strongly associated with the pneumococcal capsule thickness, and was proportional to the disease severity. Interestingly, during secondary pneumonia after primary influenza infection, intense pulmonary NETs generation together with elevated myeloperoxidase activity and cytokine dysregulation determined the disease severity. These findings highlight the crucial role played by the size of pneumococcal capsule in determining the extent of innate immune responses such as NETs formation that may contribute to the severity of pneumonia.

## INTRODUCTION

Pathogenic pneumococci are characterized by the capsule they carry on their surfaces [1]. Strong associations have been established between the thickness of pneumococcal capsule and the prevalence of nasopharyngeal carriage [2, 3]. The capsule type of the pneumococcal strain is crucial for virulence, with deletion of capsule operon exerting a profound effect on bacterial invasiveness in animal models [4, 5].

Few studies have addressed the effects of bacterial capsules on immune cell-driven processes such as the formation of neutrophil extracellular traps (NETs or NETosis). The fungal pathogen *Cryptococcus neoformans*, and its major capsule polysaccharide inhibit NETosis *in vitro*, whereas its capsule mutant and a minor polysaccharide induce NETs [6]. In another study, capsule mutants of *Burkholderia pseudomallei* induce higher levels of NETs compared to wild-type bacteria [7]. Wartha et al. [8] documented the role of capsule in the evasion of *Streptococcus pneumoniae* from NETs entrapment, but not complete protection from NETs-mediated killing. Given that neutrophils come in close contact with the outer polysaccharide of pneumococci during activation, it is imperative to understand the capsule's direct effect on neutrophil activities such as NETosis.

NETs were initially documented as part of extracellular antimicrobial mechanisms [9]. However, the presence of cytotoxic proteins such as myeloperoxidase (MPO), neutrophil elastase and histones implicate the DNA traps in many pathological conditions involving tissue damage, autoimmune and inflammatory diseases [10-12]. Indeed, using a lethal influenza animal model, we previously demonstrated the presence of NETs in close proximity to alveolar spaces and terminal bronchioles which contribute to endothelial injury [13]. Moreover, we also found that NETs are generated more frequently within murine lungs during secondary pneumococcal pneumonia following primary influenza infection. Although these NETs formed during secondary infection do not confer any significant antibacterial activity, they are associated with extensive pulmonary injury [14].

Since the capsule constitutes a critical factor that may influence neutrophil-mediated host immune responses, we investigated the influence of capsule on NETosis during both primary and secondary pneumococcal pneumonia. We used pneumococcal serotypes of varying virulence to explore the association of overall disease severity with NETosis. By comparing infections with serotypes 3, 4 and 19F in mice, we found that the thickness of capsule is directly proportional to the pulmonary NETosis and overall pathogenesis during primary pneumonia. We also found that greater degree of encapsulation improved bacterial survival against neutrophil surface killing in which non-phagocytic killing predominated. However, during pneumococcal pneumonia secondary to influenza, pulmonary NETosis and pathogenesis were not influenced exclusively by capsule size. This indicates the role of other pneumococcal virulence mechanisms during disease progression after influenza-induced damage. Furthermore, a capsule mutant of serotype 4 induced significantly less pulmonary NETs compared to its wild-type counterpart, and was found to be incompetent in causing clinical manifestations in secondary infected animals. Our findings suggest that the pneumococcal capsule contributes to pulmonary NETs formation, and drives the pathogenesis of capsule-enriched serotypes in healthy hosts, while prior influenza infection can augment other pneumococcal factors (besides capsule) to instigate NETosis.

## RESULTS

### Purified pneumococcal capsular polysaccharide induces NETs in a dose-dependent manner

**Figure 1:**
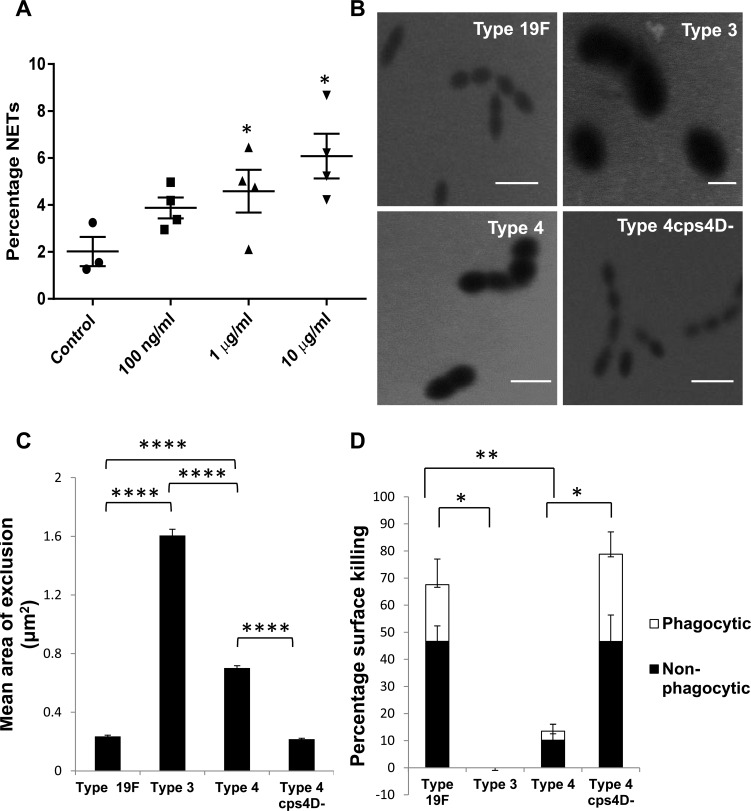
Capsule polysaccharide induces NETs and protects pneumococci from neutrophil-mediated surface killing **A.** Bone marrow-derived neutrophils were stimulated for 2 h with various concentrations of purified capsule polysaccharide from serotype 4 (x-axis). Percentage NETs data (y-axis) are presented as means ± SE (*n* = 3 independent experiments per group). The purified capsule polysaccharide induced NETs in a concentration-dependent manner. *indicates *P* value < 0.05. **B.** FITC-dextran (2000 kDa) exclusion assay was performed to estimate the capsule size of pneumococcal strains. Representative images from FITC-dextran exclusion assay. Scale bars = 2 μm. **C.** Serotype 3 had the largest capsule (∼8-fold > 19F) followed by serotypes 4 and 19F. Serotype 4 capsule mutant had significantly smaller (∼3-fold) capsule than its wild-type. *n* = 100 bacterial cells per strain. **D.** Serotype 3 was completely resistant to neutrophil-mediated killing, whereas serotype 19F was easily killed (almost 70%). Serotype 4 was killed to a lesser extent (∼10%), while its capsule mutant (4cps4D- lacking the *cps4D* gene) was highly susceptible to killing (∼80%). For all strains, non-phagocytic killing predominated over phagocytic killing. Data are presented as means ± SE (*n* = 3 per strain). *indicates *P* < 0.05, **P < 0.01, ****P < 0.0001.

We previously demonstrated increased NETs generation following pneumococcal superinfection compared to primary influenza infection [14]. Since bacterial surface components are the first to come in direct contact with phagocytes, we studied the effect of capsular polysaccharide on NETs. To determine if encapsulated bacterial polysaccharide by itself can induce NETs, we incubated bone marrow-derived neutrophils with capsular polysaccharide purified from *S. pneumoniae* serotype 4 for 2 h. We observed significant induction of NETs that increased with the polysaccharide concentration, with 10 μg/ml inducing 3-fold greater NETs than control (Figure 1A).

### Capsule size of pneumococci determines their susceptibility to neutrophil-mediated killing

Strains representing three serotypes of *S. pneumoniae* were compared for their vulnerability to surface killing by neutrophils. Serotypes 3, 4 and 19F are clinically prevalent strains with global distribution exhibiting different degrees of invasiveness [15]. All three serotypes are encapsulated as confirmed by India ink staining technique (Supplementary Figure 1A). To exclude non-capsular factors, we included a mutant of serotype 4 (designated 4cps4D-) that produces less polysaccharide than its wild-type counterpart (Supplementary Figure 1B).

To estimate the capsule size, FITC-dextran exclusion assay was performed and the zone of dextran exclusion was measured. Serotype 3 possessed the largest capsule, followed by serotype 4, while serotype 19F had the smallest capsule. As expected, the serotype 4 capsule mutant had a significantly smaller capsule, approximately 30% of its wild-type counterpart (Figure 1B and 1C).

To determine the importance of the capsule in evading neutrophil-mediated killing, we subjected the pneumococcal strains to surface killing assays. We found non-significant killing of serotypes 3 and 4 by neutrophils (∼0-10%), while serotype 19F and capsular mutant 4cps4D- were killed effectively (∼70-80%), as indicated in Figure 1D. This reveals an association between capsule thickness and the susceptibility of *S. pneumoniae* to neutrophil-mediated killing. Interestingly, non-phagocytic killing (including by NETs) predominated over phagocytic killing. However, compared to its wild-type, 4cps4D- displayed relatively greater vulnerability to phagocytic killing (i.e. ∼10-fold increase of phagocytic killing *versus* ∼6-fold increase of total killing).

### Capsule thickness correlates with overall pathogenesis and pulmonary NETosis during primary pneumococcal pneumonia

**Figure 2:**
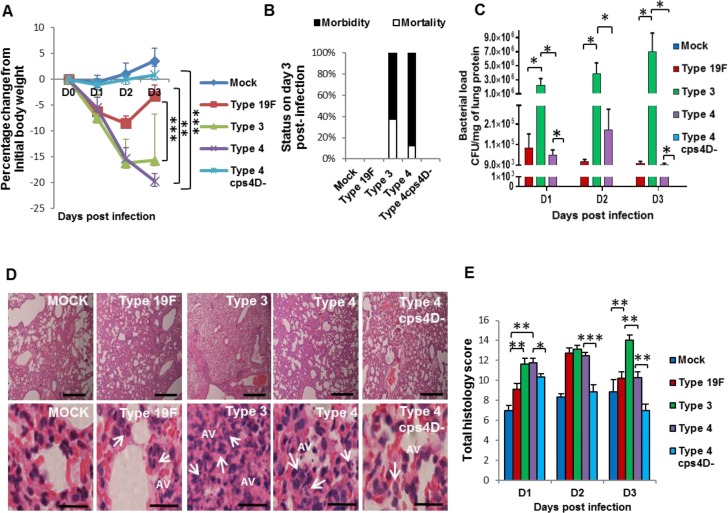
Capsule thickness correlates with pneumococcal virulence during primary lung infection in mice BALB/c mice were intratracheally infected with 10^7^ CFU of serotypes 19F, 3 and 4 as well as the capsule mutant of type 4 (4cps4D-). Lungs were harvested on 1, 2 and 3 days post-infection (dpi). **A.** Infection with both serotypes 3 and 4 caused significant body weight loss until 3 dpi, whereas serotype 19F recovered by 3 dpi, while 4cps4D- failed to cause any clinical symptoms. **B.** Both serotypes 3 and 4 induced 100% morbidity, with type 3 causing higher mortality. Type 19F and 4cps4D- did not cause any morbidity. **C.** Primary infection of mice with serotype 3 led to the highest bacterial load in the lungs. Serotype 4 (wild-type) exhibited bacterial load, whereas its capsule mutant was absent in the lungs. **D.** Hematoxylin and eosin staining of lung sections revealed considerable neutrophil infiltration (arrows) in the alveolar spaces (AV) arising from infection with types 3, 4 and 19F. Mock control and 4cps4D- displayed the least neutrophil presence in lungs. Magnification and scale bars: top panels = 100× & 500 μm; lower panels = 1000× & 25 μm. **E.** Histopathologic scoring revealed the worst lung pathology caused by serotype 3, while types 19F, 4 and 4cps4D- showed less infiltration by 3 dpi. Data are presented as means ± SE (*n* = 8 per group). *indicates *P* < 0.05, **P < 0.01, ***P < 0.001.

To elucidate the influence of pneumococcal capsule during primary infection of lungs, we intratracheally challenged mice with a high dose (10^7^ colony-forming units or CFU) of the four bacterial strains, and monitored them for three consecutive days. Mice infected with all wild-type strains displayed body weight loss, whereas those infected with 4cps4D- did not show any noticeable change (Figure 2A). Mice infected with serotypes 3 and 4 displayed severe clinical manifestations such as drastic weight loss, breathlessness, morbidity by day 3, whereas mice with serotype 19F recovered on day 3. Serotype 3 culminated in higher mortality (37.5%) than serotype 4 (12.5%), although both groups eventually had to be euthanized on day 3 due to severe morbidity (Figure 2B). Lung bacterial loads (Figure 2C) were significantly higher for serotype 3 (e.g. >5×10^6^ CFU/g on day 3) than serotypes 4 and 19F (e.g. ∼9×10^3^ CFU/g on day 3). This corresponds well with the *in vitro* data (Figure 1D) indicating that serotype 3 was resistant to neutrophil-mediated killing, and could thus replicate uninhibited. Mutant 4cps4D- colonies could not be recovered from the lungs on all days, implying very low *in vivo* replication rate and/or clearance by immune cells (Figure 2C).

Upon histopathologic examination, serotype 3 infection was the most severe (followed by serotype 4) causing considerable necrosis, edema and cellular infiltration in the lungs (Figure 2D and 2E). High accumulation of neutrophils was evident in alveolar-capillary spaces across all wild-type infections, whereas the capsule mutant portrayed a mixed cellular response on day 1 with very minimal infiltration thereafter (Figure 2D). Infections with serotypes 3 and 4 showed marked cellular infiltration on days 1 and 2. By day 3, serotype 3 revealed the progressively worst pathology, while serotypes 4 and 19F had decreased scores. On days 2 and 3, the capsule mutant exhibited significantly lower scores, e.g. ∼30% less than serotype 4 on day 3 (Figure 2E).

**Figure 3:**
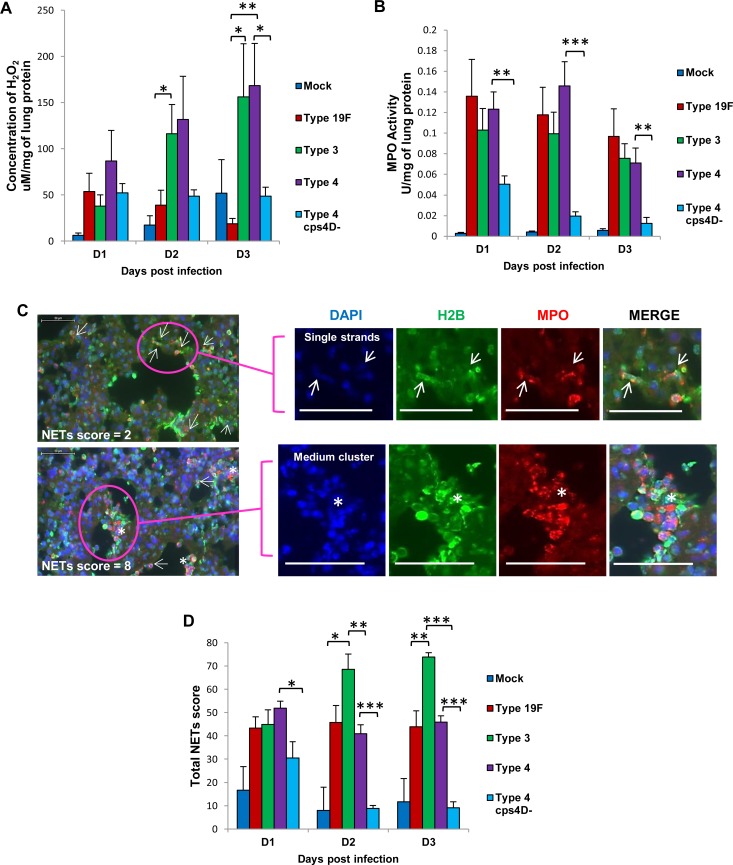
NETs score, but not H2O2 or MPO activity, correlates with overall virulence after primary pneumococcal lung infection **A.** Serotype 4 induced the highest H_2_O_2_ concentration in the lungs of infected mice. **B.** MPO activity partially corresponded with H_2_O_2_ concentration, and was the lowest after serotype 4 mutant infection. **C.** NETs were identified and scored in the lung sections by triple immunolabeling (DAPI = blue, histone H2B = green, MPO = red). Arrows indicate single NET strands, whereas asterisks represent clusters. Scale bars = 50 μm. **D.** NETs score correlated with capsule thickness and clinical severity. Serotype 3 induced the highest NETs at 2 and 3 dpi. Infection with 4cps4D- was significantly lower than its wild-type for all parameters. Data are presented as means ± SE (*n* = 8 per group). *indicates *P* < 0.05, **P < 0.01, ***P < 0.001.

Upon assessing the reactive oxygen species (ROS) concentration and neutrophil activity in the lungs, we found the highest levels of hydrogen peroxide (H_2_O_2_) in mice infected with serotype 4, followed by serotype 3. Serotype 19F and 4cps4D- infections had significantly lower H_2_O_2_ levels in lungs, e.g. about 3- to 6-fold less than serotypes 3 and 4 on day 3 (Figure 3A). MPO activity was generally elevated for all wild-type infections compared to capsule mutant (Figure 3B).

Formation of NETs in the lung sections of infected mice correlated well with the histopathologic severity during serotype 3 infection, showing the highest occurrence of pulmonary NETs on day 3, i.e. almost double those for serotypes 19F and 4 (Figure 3C and 3D). Consistent with our observation that purified polysaccharide could induce NETs (Figure 1A), we found that *in vivo* infection with the capsule mutant generated significantly less NETs, e.g. over 4-fold lower score than its wild-type on day 3 (Figure 3D), thus indicating that pneumococcal capsular polysaccharides contribute substantially to NETs formation during pathogenesis. The correlation of NETs with histopathologic severity suggests the histopathologic relevance of NETs in pneumococcal pneumonia. This is congruent with the similar association between NETs and severe pathology noted during primary influenza virus pneumonia [13].

### Capsule in concert with other factors contribute to pathogenesis during secondary pneumococcal pneumonia following primary influenza

**Figure 4:**
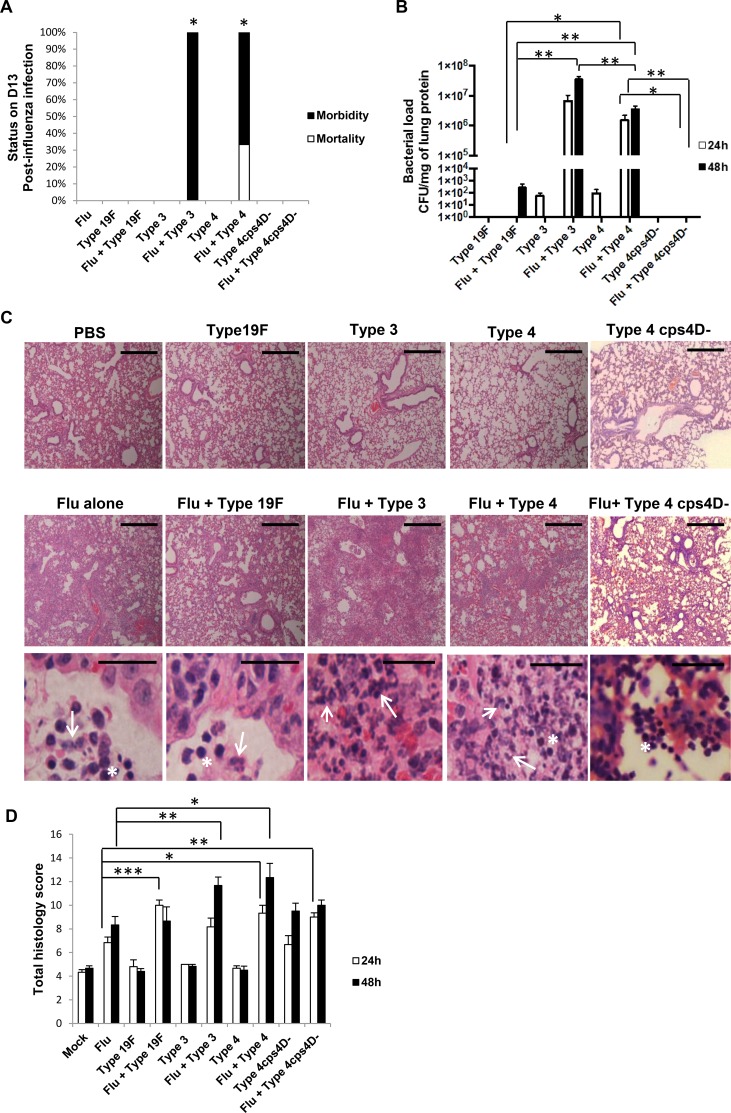
Capsule thickness alone does not determine the severity of secondary pneumococcal pneumonia after primary influenza infection BALB/c mice were first intratracheally infected with 5 PFU of PR8 influenza virus (sub-lethal dose), followed by 100 CFU of *S. pneumoniae* after 7 days. Mice were monitored daily until they lost 30% of initial body weight or were euthanized at specific time-points. Lungs were harvested at 24 and 48 h after secondary infection. **A.** Secondary infections with both serotypes 3 and 4 led to 100% morbidity by day 10, while mice in other groups survived until the experimental end-point. Kaplan-Meier analyses revealed that survival rates of secondary infections with types 3 and 4 were statistically lower than influenza alone, and secondary infections with types 19F and 4cps4D-. *n* = 3 per group. *represents *P* < 0.05. **B.** All the serotypes replicated efficiently in the lungs during secondary infection (after influenza) compared to bacteria infection alone. Secondary infections with serotype 3 followed by serotype 4 led to the highest pulmonary bacterial loads. However, the capsule mutant was not recovered from the lungs. **C.** Hematoxylin and eosin staining of lung sections revealed intense neutrophil infiltration (arrows) in the alveolar spaces of secondary infected groups of serotypes 3, 4 and 19F. Notably, secondary infection with 4cps4D- showed strong inflammatory cell infiltration, comprising mostly lymphocytes (asterisks). Magnification and scale bars: 100× & 500 μm (top two panels); 1000× & 25 μm (bottom panel). **D.** Histopathologic scoring revealed that secondary infections with serotype 4 (followed by serotype 3) suffered from the worst lung pathology. Data are presented as means ± SE (*n* = 6 per group). *denotes *P* < 0.05, **P < 0.01, ***P < 0.001.

To extend the correlation between capsule size and pneumococcal virulence to secondary pneumococcal pneumonia, we first infected mice with a sub-lethal dose of influenza virus for 7 days, followed by sub-lethal doses of the four bacterial strains (100 CFU each), and monitored their lungs at 24 and 48 h after pneumococcal challenge. All influenza-infected mice and those co-infected with *S. pneumoniae* exhibited similar body weight loss trends (Supplementary Figure 2). However, the mice co-infected with serotypes 3 and 4 suffered severe clinical manifestations and were moribund by days 9 or 10 post-influenza infection (Figure 4A). Hence, 48 h post-secondary infection (or day 9) was considered as the end-point for further experiments.

Consistent with previous reports [16-18], the secondary wild-type pneumococcal infections after a preceding influenza challenge greatly enhanced the replicative efficiency of *S. pneumoniae* in lungs. Bacterial load in the lungs was highest with secondary serotype 3 infection (∼10^7^ CFU/g), similar to primary pneumococcal pneumonia caused by a much higher challenge dose (Figures 4B and 2C). This was followed by secondary serotype 4 infection (∼10^6^ CFU/g), while the lung bacterial burden of secondary 19F infection was considerably much lower (∼10^2^ CFU/g). Colonies were undetectable in the lungs of secondary 4cps4D- infection (Figure 4B).

Influenza alone infection and dual-infected groups revealed more severe histopathology compared to “bacteria alone” or “mock” infections (Figure 4C and 4D). Interestingly, secondary infection with serotype 4 showed the most severe pathologic features such as increased cellular infiltration, necrosis and pleuritis, followed closely by secondary serotype 3 infection, despite the latter bearing higher lung bacterial burden. Furthermore, compared to “influenza alone” infection, secondary infection with capsule mutant resulted in significantly greater pathologic score despite being undetectable in the lungs, indicating that non-capsulated strains can also prove damaging to already compromised influenza-infected lung tissues. Intriguingly, while secondary wild-type infections caused intense pulmonary neutrophil infiltration, secondary infection with the capsule mutant showed relatively higher frequency of infiltrating lymphocytes in the lungs (Figure 4C).

**Figure 5:**
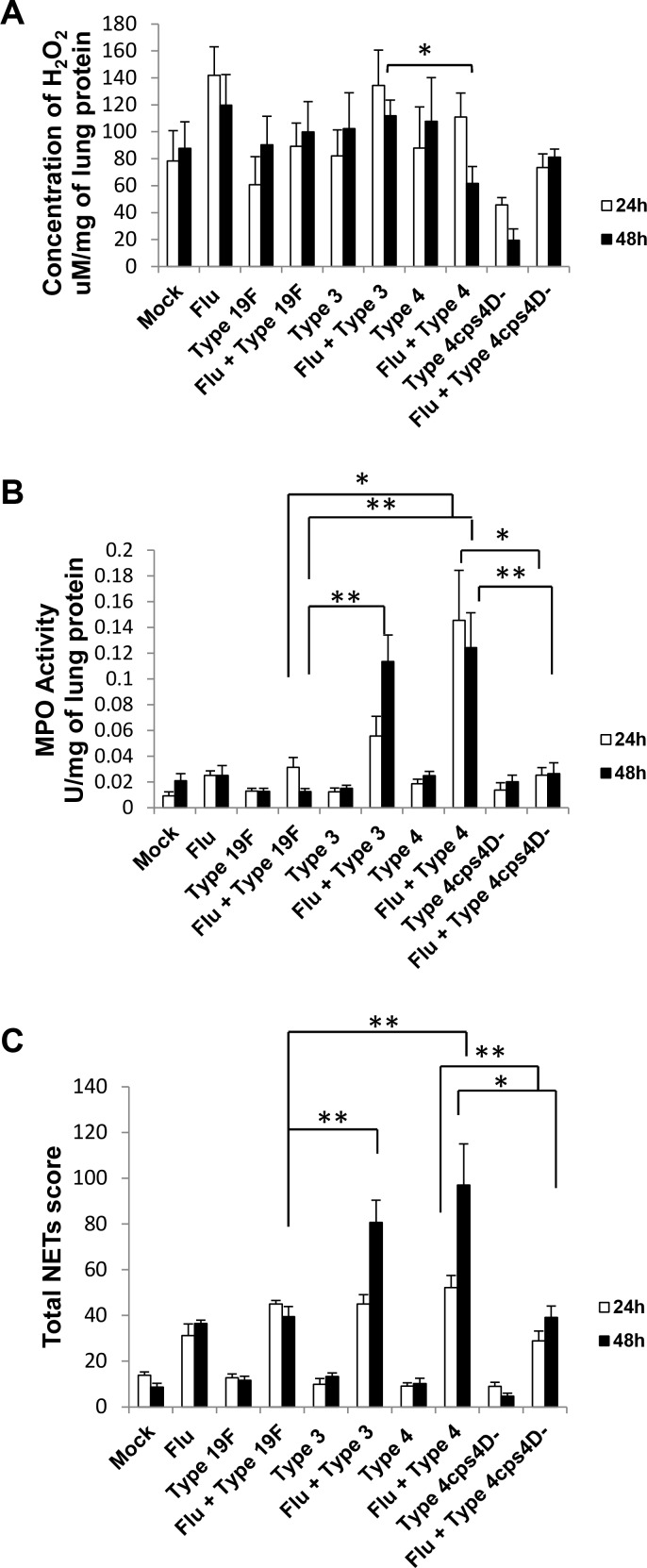
NETs and neutrophil activity in lungs generally correspond to disease severity after secondary pneumococcal infection **A.** Lung H_2_O_2_ levels were generally elevated after secondary infection especially with serotype 3. **B.** Secondary infections with serotype 4 (followed by serotype 3) exhibited the greatest MPO activity in lungs. **C.** Secondary infections with serotype 4 (followed by serotype 3) also induced the highest pulmonary NETs score. Secondary infection with 4cps4D- resulted in significantly lower NETs and MPO activity than its wild-type. Data are presented as means ± SE (*n* = 6 per group). *P < 0.05, **P < 0.01.

The lung ROS content as reflected by H_2_O_2_ concentration showed generally elevated levels after secondary infection with the three serotypes, but without striking differences (Figure 5A). In contrast, secondary infection with serotype 4 revealed the highest MPO activity (∼5 to 10-fold higher than secondary infection with serotype 19F), congruent with its histopathologic severity (Figure 5B). Secondary infection with serotype 3 exhibited the next highest MPO activity. Notably, compared to its wild-type, secondary infection with the capsule mutant showed significantly lower MPO activity, i.e. ∼5 to 6-fold lower (Figure 5B).

NETs formation mirrored the patterns of histopathology and MPO activity, with secondary serotype 4 infection inducing the greatest NETs formation in the lungs followed by secondary serotype 3 infection, i.e. ∼2.5-fold and ∼2-fold higher than secondary 19F infection at 48 h respectively (Figure 5C). The NETs score for secondary capsule mutant infection was less than half that of its wild-type counterpart, further reiterating the importance of capsule in triggering NETs formation.

**Figure 6:**
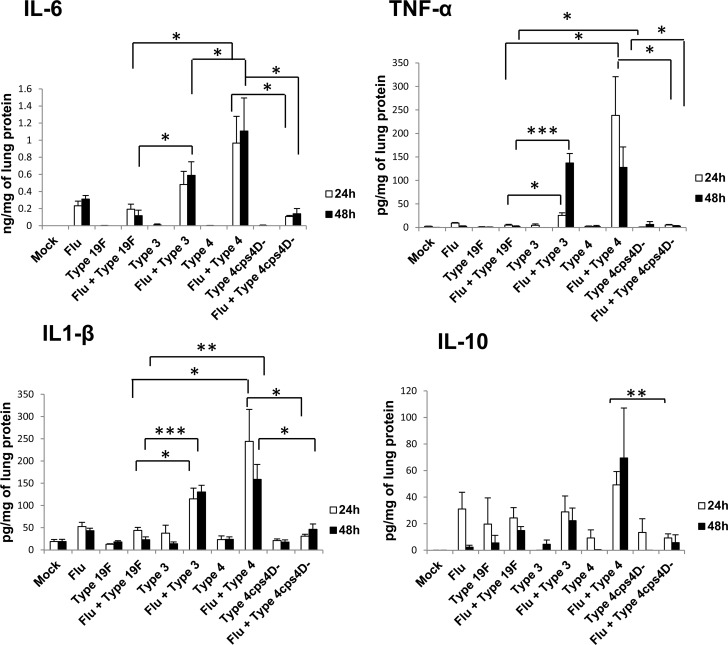
Secondary infection with virulent pneumococci induces augmented levels of pro-inflammatory cytokines in lungs Secondary infections with serotype 4 (followed by serotype 3) elicited the highest cytokine levels. IL-17 was not detectable in the lung homogenates. Data are presented as means ± SE (*n* = 4 per group). *indicates *P* < 0.05, **P < 0.01, ***P < 0.001.

Cytokine storm induced by immune dysregulation is implicated in severe influenza epidemics such as the 1918 “Spanish flu” [19, 20]. Pro-inflammatory cytokines such as IL-8 are potent inducers of NETs [9] that can contribute to tissue injury. MAP kinase phosphatases (MKPs) are negative regulators of cytokine expression, and exert profound effects on innate immunity especially after influenza infection [21-23]. To understand the association between inflammatory cytokines and NETs *in vivo*, we analyzed the mRNA expression of pro-inflammatory cytokines and MKPs in lung homogenates (Figure S3). Expression levels of *IFN-β* and *IL-1β* were highest in secondary infection with serotype 3, followed by secondary serotype 4 infection. Increased expression of *RANTES* relative to mock-infection was observed in the “influenza only” infection group as well as in all secondary infections, indicating its importance in host response to influenza virus. Protein concentrations of IL-6, IL-1β, TNF-α and IL-10 (Figure 6) were consistently the highest in secondary infections with serotype 4 followed by serotype 3. On the other hand, secondary infection with capsule mutant culminated in significantly lower levels of these cytokines than its wild-type counterpart. IL-17 was not detectable in all samples. Selected MKP genes were also analyzed to ascertain the expression profiles of these cytokine-regulatory factors. *MKP-2* mRNA expression was generally enhanced after “influenza only” infection and secondary pneumococcal infections. In contrast, *MKP-5* mRNA expression was generally lower for secondary infections, compared to primary infections with all four pneumococcal strains at 48 h. Interestingly, *MKP-3* expression was relatively reduced at 48 hours after primary and secondary infections with serotypes 3 and 4, indicating *MKP-3* dysregulation by infection with virulent pneumococci (Supplementary Figure 3).

We also performed both primary and secondary pneumococcal infections *in vitro* using bronchoalveolar lavage fluid (BALF) from influenza-infected mice. All infectious conditions including only influenza-infected BALF (Flu) induced higher percentages of NETs than unstimulated neutrophils (Control). Stimulation of bone-marrow derived neutrophils *in vitro* using *S. pneumoniae* strains culminated in a pattern of NETs induction somewhat different from our *in vivo* observations of NETosis (Supplementary Figure 4). Thus, the greatest extent of NETosis *in vitro* was generated by secondary infections with serotype 19F and 4, and by primary infections with serotype 3 and capsular mutant.

## DISCUSSION

In this study, we explored the differences between three pneumococcal serotypes in inducing NETs based on their capsule size. We found that the degree of NETs induction and virulence was proportional to capsule thickness. Serotype 3 had the thickest capsule, was resistant to neutrophil-mediated killing, and induced the highest extent of pulmonary NETs after primary infection. This was followed by serotypes 4 and 19F. Moreover, a capsule mutant of serotype 4 (4cps4D-) possessed a much thinner capsule, and was highly susceptible to neutrophil-mediated killing that rendered it avirulent in mice. However, secondary pneumococcal infection following primary influenza did not completely correlate with capsule size. While serotype 3 persisted in the lungs at the highest concentrations, serotype 4 induced the greatest pulmonary NETs scores and the most severe disease. In secondary infection, the capsule mutant did not survive within the lungs, but stimulated intense infiltration of lymphocytes into the lungs.

The pneumococcal capsule facilitates the bacteria to evade entrapment by NETs, even though the diplococci can also degrade the NETs structures using DNase [8, 24]. Although earlier reports indicate an inhibitory effect of some types of capsular polysaccharide on NETs [6, 7], here we demonstrate for the first time that pneumococcal capsular polysaccharide by itself can induce NETs in a dose-dependent manner. Such differences amongst capsule polysaccharides may be attributed to the biochemical composition of individual polysaccharides of pathogens from different backgrounds. Additionally, reduction of capsule polysaccharide in serotype 4 favored phagocytosis against NETs, indicating the distinct influence of the capsule on neutrophil function, e.g. its role in evading phagocytosis. The capsule mutant was also incapable of establishing efficient infection *in vivo* suggesting that the pneumococcal capsule constitutes a key factor that determines bacterial virulence as well as the mode of host neutrophil responses.

During primary pneumococcal pneumonia, capsule size played a vital role in the pathogenesis since it correlated with disease progression and pulmonary NETs formation. However, this correlation with capsule thickness was not fully obvious during secondary pneumococcal pneumonia, suggesting additional pathogen and host factors that may govern the severity of secondary infection. Pneumococci can readily multiply in high numbers within airways that are already damaged and compromised by primary influenza infection [14, 25]. However, each serotype may harness unique mechanisms to explain the pathological differences between serotypes. Studies using serotypes 3, 4 and 19F reveal that serotype 4 generates the highest H_2_O_2_ under aerobic conditions, and hence causes the highest frequency of DNA double-stranded breaks in infected lungs [26]. Although no serotype-specific differences in pulmonary H_2_O_2_ levels during secondary infections were observed, secondary infection with serotype 4 (followed by serotype 3) resulted in the highest MPO activity and the greatest NETs induction. These findings allude to other virulence mechanisms such as pneumolysin, or uptake of pneumococci assisted by platelet-activating factor receptor which undergoes rapid internalization upon binding to a ligand. This property is a feature of pneumococcal virulence since non-virulent strains do not adhere efficiently *via* this mechanism [27].

Despite the difference in correlation with serotype virulence, the capsule remained crucially important in exerting the NETs-inducing and pathogenic capability of pneumococci. During primary and secondary pneumonia, the serotype 4 capsule mutant producing much reduced polysaccharide failed to cause significant pneumococcal disease manifestation, lung pathology nor NETs, in marked contrast to its wild-type. This is congruent with earlier reports showing that loss of capsular genes or loci have a negative effect on pneumococcal colonization and invasion in mice [28-30].

Intriguingly, secondary infection by the capsule mutant led to greater lymphocyte accumulation in the lungs as opposed to neutrophil infiltration by the wild-type. Given that primary infection with the capsule mutant did not display such strong predilection for lymphocytes, it is not very clear whether and how the capsule can dictate myeloid/lymphoid cell activation, possibly through cytokine signaling. Nevertheless, this observation strengthens the widely held notion that the capsule of *S. pneumoniae* qualitatively determines the host immune response.

Interestingly, *in vitro* studies using bone-marrow derived neutrophils did not yield similar findings as *in vivo* experiments. This discrepancy may be explained by the limited bacteria-neutrophil interactions under *in vitro* conditions. *In vivo*, however, neutrophils and pathogens come together in contact in an environmental milieu influenced by overlapping factors, such as pro-inflammatory chemokines and ROS, both of which stimulate neutrophils to induce NETosis *via* multiple receptors [9, 31, 32].

In this study, we have investigated three relatively prevalent serotypes of pneumococcus that are included in the pneumococcal polysaccharide vaccines [33]. The capsule sizes of all the pneumococcal serotypes have not yet been determined. All serotypes (except types 3 and 37) utilize similar capsule biosynthesis pathways [5]. However, there may be clonal differences within serotypes with varying invasive potential that may elicit qualitatively and/or quantitatively different immune responses, including NETosis [34]. Hence, further studies using the *in vivo* mouse infection models are warranted to test each pneumococcal serotype individually to elucidate its potential to inflict pulmonary injury, induce NETosis and other parameters.

In conclusion, we demonstrate that the pneumococcal capsule exerts significant influence on NETosis and lung injury during primary and secondary pneumonia in mice. The pneumococcal capsule directly contributes to pulmonary NETosis that correlates with disease severity in mice during primary pneumonia. In contrast, during secondary pneumonia following influenza, the capsule in concert with other pneumococcal virulence factors drive pulmonary NETosis and disease pathogenesis.

## MATERIALS AND METHODS

### Animals and ethics

All experiments involving animals were performed according to the regulations of Institutional Animal Care and Use Committee, National University of Singapore (protocol number 050/11). Female BALB/c mice (7-10 weeks old) were housed in micro-isolator cages in a BSL-2 animal facility.

### Virus and bacterial strains

Influenza virus and *S. pneumoniae* were cultured as described previously [14]. Influenza virus A/Puerto Rico/8/34 H1N1 strain (PR8) was obtained from the American Type Culture Collection (ATCC), propagated in embryonated eggs, and the viral titers were determined by plaque assay. *S. pneumoniae* serotypes 3 (A66.1 Xen 10), 4 (TIGR), and 19F (clinical isolate from Singapore), were cultured till mid-logarithmic phase in brain-heart infusion broth (Sigma) supplemented with 5% heat-inactivated fetal bovine serum (FBS) under anaerobic conditions. Type 4cps4D-, a capsule mutant of serotype 4 that lacks the *cps4D* gene (Supplementary Figure 1B) was generated previously [35], and was cultured under the same conditions as its wild-type counterpart.

### Measurement of pneumococcal capsule thickness

Capsule thickness was measured as the zone of exclusion of FITC-dextran as described previously [3]. *S. pneumoniae* at mid-logarithmic phase (20 μl) was mixed with 2 μl of 2000 kDa FITC-Dextran (10 mg/ml stock; Sigma), and wet-mounts were prepared on glass slides. The preparations were visualized using an Olympus IX81 microscope equipped with FV10-ASW 3.0 viewer. Images were captured at 3000× magnification from two independent experiments, and the zones of dextran exclusion were estimated using ImageJ software. The areas of 100 bacterial cells were measured, and the mean area (μm^2^) was calculated to represent capsule thickness.

### Isolation of bone marrow-derived neutrophils and induction of NETs

Neutrophils were isolated from healthy mice using Percoll gradient, and NETs were induced as described previously [14]. Briefly, 10^5^ cells each were seeded onto poly-L-lysine-coated 8-well chamber slides, the required stimulus was added in RPMI-1640 medium, and incubated for 2 h at 37°C. Purified pneumococcal polysaccharide of serotype 4 (34-X, ATCC) was added at 100 ng, 1 μg and 10 μg concentrations. *S*. *pneumoniae* serotypes 19F, 3, 4, and 4cps4D- were added at multiplicity of infection (MOI) of 1 for primary induction of NETs. To simulate secondary infection, neutrophils were first incubated with BALF collected on day 5 after infection of mice with 500 plaque-forming units (PFU) of PR8 virus (lethal dose). After 2 h, *S*. *pneumoniae* were added at MOI of 1, and incubated for 2 h. The cells were then fixed, and stained with rabbit polyclonal antibody to MPO (Abcam) and mouse monoclonal antibody to histone H2B (Abcam) along with DAPI. The slides were examined by fluorescence microscopy at 4000× magnification. For each sample, NETs were counted within at least 10 fields, and represented as percentage of total neutrophils. Full details are outlined in Supplementary Information.

### Surface killing assay

Based on a modified protocol [3], surface killing assays were performed with replicates for each condition. Mid-logarithmic *S. pneumoniae* cultures were adjusted to 4×10^3^ CFU/ml in RPMI-1640 medium, 10 μl of each culture were spotted on blood agar plates, and allowed to dry. At least 4-6 spots were placed per plate. Neutrophils (4×10^4^) were overlaid onto each spot, and allowed to dry. To inhibit phagocytosis, 10 μg/ml of cytochalasin B was incubated with neutrophils for 30 min prior to use. The plates were then incubated overnight at 37°C under anaerobic conditions. The average number of colonies per condition was calculated, and then expressed as the percentage of phagocytic and non-phagocytic killing over total killing (i.e. Neutrophils + Bacteria = Total killing; Neutrophils + Cytochalasin B + Bacteria = Non-phagocytic killing; Total killing - Non-phagocytic killing = Phagocytic killing).

### Primary and secondary pneumococcal infections of mice

Mice were anesthetized using 75 mg/kg ketamine and 1 mg/kg medetomidine. For primary pneumococcal infection (*n* = 8), 10^7^ CFU bacteria were intratracheally instilled in mice with phosphate-buffered saline (PBS). For secondary infection (*n* = 6), mice were first intratracheally infected with a sub-lethal dose of PR8 influenza virus (5 PFU), followed by intratracheal challenge with *S. pneumoniae* (100 CFU). Control mice received PBS alone. The anesthesia was reversed using atipamezole hydrochloride (5 mg/ml, 0.1 ml per 10 g). The mice were euthanized on specified days, their lungs were excised, with one lobe fixed in 4% paraformaldehyde, while the other lobe was snap-frozen for later assays.

### Histopathologic analyses

Formalin-fixed lungs were dehydrated, embedded in paraffin; sections (5 μm) were prepared, and stained with hematoxylin and eosin for histopathologic scoring. Multiple fields were analyzed per sample in a blinded manner based on modified criteria [36]. Cellular infiltration, necrosis, pleuritis, and fibrin deposition were scored from 0 to 3 where: 0 = absent; 1 = mild, 2 = moderate; and 3 = severe. The percentage of affected lung parenchyma was scored as: 0 = no area affected; 1 = <10%; 2 = 10-30%; and 3 = >30% of lung surface, respectively. The final injury score was added according to the formula: cellular infiltration + necrosis + pleuritis + fibrin + percentage of lung involvement.

### Quantification of NETs in lung sections

NETs were stained as described previously [14]; lung sections were deparaffinized, permeabilized and stained with antibodies against histone H2B and MPO together with DAPI. NETs were scored in 20 fields from each whole section using predetermined criteria based on their morphologic appearance as individual strands or clusters (Figure 3C, Supplementary Figure 5, and Supplementary Table 1). Full details are outlined in Supplementary Information.

### Bacterial load in mouse lungs

Frozen mouse lung tissues were homogenized using the gentleMACS homogenizer (Miltenyi Biotec). Aliquots of homogenates were serially diluted, plated on blood agar, and colony counts were determined by the spread plate method.

### H_2_O_2_ concentration and MPO activity assays

Fresh lung homogenate aliquots were tested in replicates to estimate H_2_O_2_ concentration by the Amplex red hydrogen peroxide/peroxidase assay kit (Invitrogen). MPO activity was determined using a modified method [37]. Lung homogenate (10 μl) was mixed with 190 μl of freshly-prepared assay solution (26.9 ml H_2_O; 2.0 ml 0.1 M sodium phosphate buffer, pH 7.0; 0.1 ml 0.1 M H_2_O_2_; and 0.048 ml guaiacol) in 96-well plates at room temperature, and the absorbance was read immediately at 470 nm for 1 min using Infinite M200 multimode reader (Tecan). MPO activity was calculated as Units/ml = (ΔO.D. × V_t_ × 4) / (E × Δ_t_ × V_s_) × 2 where V_t_ = total volume (ml), V_s_ = sample volume (ml), ΔO.D. = optical density change, Δ_t_ = time of measurement (minutes), and 2 is the conversion factor to 1-cm path-length. Four moles of H_2_O_2_ are required to produce 1 mole of tetraguaiacol product which has the extinction coefficient (E) of 26.6 mM^−1^ cm^−1^ at 470 nm. All values were normalized to lung protein content as measured by the Bradford method (Bio-Rad).

### RNA extraction and real-time PCR analyses

RNA samples were extracted from mouse tissues using TRIzol (Invitrogen), and converted to cDNA using ImProm-II Reverse Transcription system (Promega). Each cDNA was subjected to quantitative real-time PCR with SYBR Green PCR master mix using a CFX Connect Real-Time PCR Detection system (Bio-Rad). The relevant PCR primers are listed in Supplementary Table 2. Relative gene expression levels were calculated using the 2^−ΔΔCt^ formula.

### Enzyme-linked immunosorbent assays (ELISA)

Sandwich ELISA was performed to determine the concentrations of TNF-α, IL-6, IL-10 and IL-17 using corresponding antibodies (BD Pharmingen). IL-1β concentration was measured using an ELISA kit (R&D Systems) according to the manufacturer's protocol.

### Statistical analyses

Statistical analyses were performed using SPSS (version 22). Student's *t*-test was used for pairwise comparison. ANOVA with Tukey post-hoc correction were used for comparison of more than two groups. *P* values less than 0.05, 0.01, 0.001 and 0.0001 were considered significant (to varying degrees).

## SUPPLEMENTARY MATERIAL FIGURES AND TABLES


