# Supplementary material for: Capsules of virulent pneumococcal serotypes enhance formation of neutrophil extracellular traps during in vivo pathogenesis of pneumonia
Source: Oncotarget. 2016 Mar 28;7(15):19327–40. doi: 10.18632/oncotarget.8451 (PMC4991386; doi:10.18632/oncotarget.8451)
Supplement: Supplementary file 1 [file oncotarget-07-19327-s001.pdf]

Capsules of virulent pneumococcal serotypes enhance formation of neutrophil extracellular traps during *in vivo* pathogenesis of pneumonia

Supplementary Material

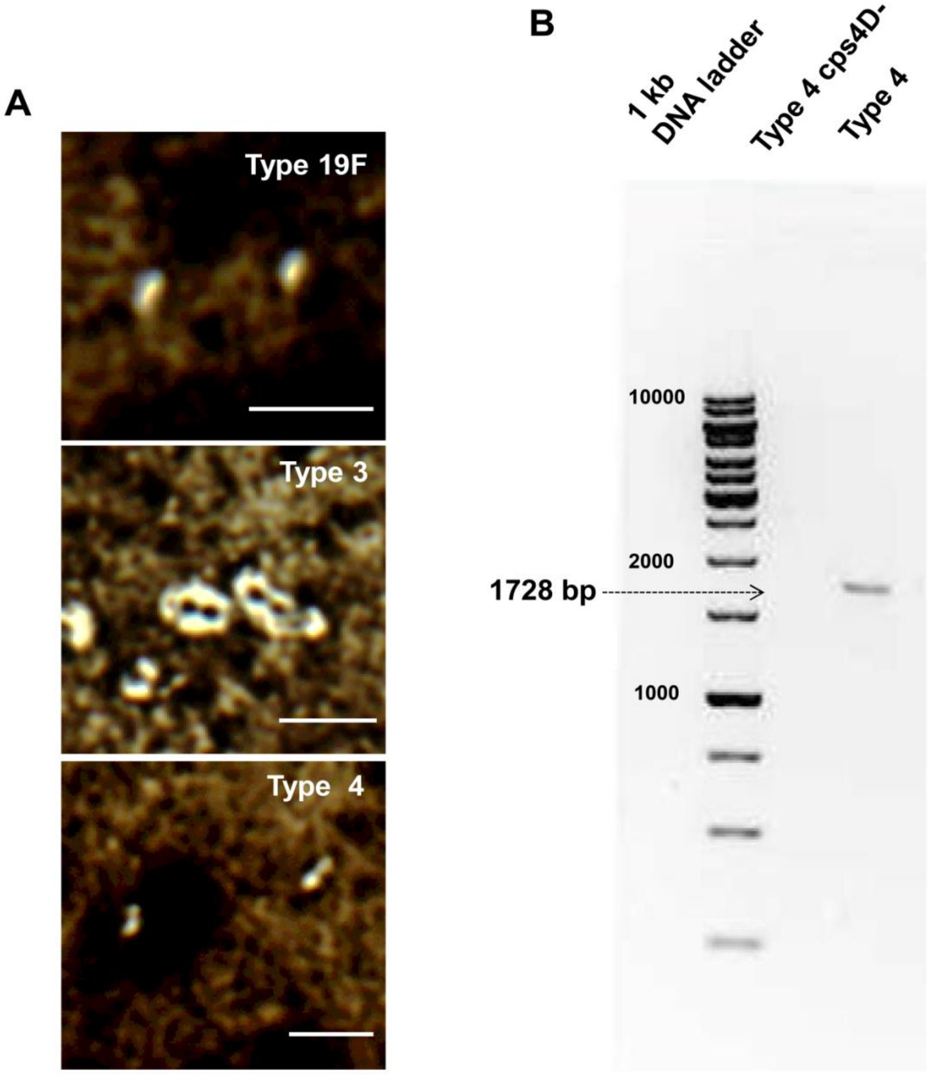

Supplementary Figure 1: All wild-type pneumococci possess capsules while type 4cps4D- lacks the *cps4D* gene. A. Capsules of pneumococcal serotypes 3, 4 and 19F were visualized using India ink stain. Serotype 3 possessed the largest capsule seen as a thick halo surrounding the bacterial cells. Representative images are shown. Scale bars = 4 µm. **B.** PCR amplification of *cps4D* confirmed the absence of the polysaccharide export gene in the 4cps4D- mutant strain, which is present in the wild-type.

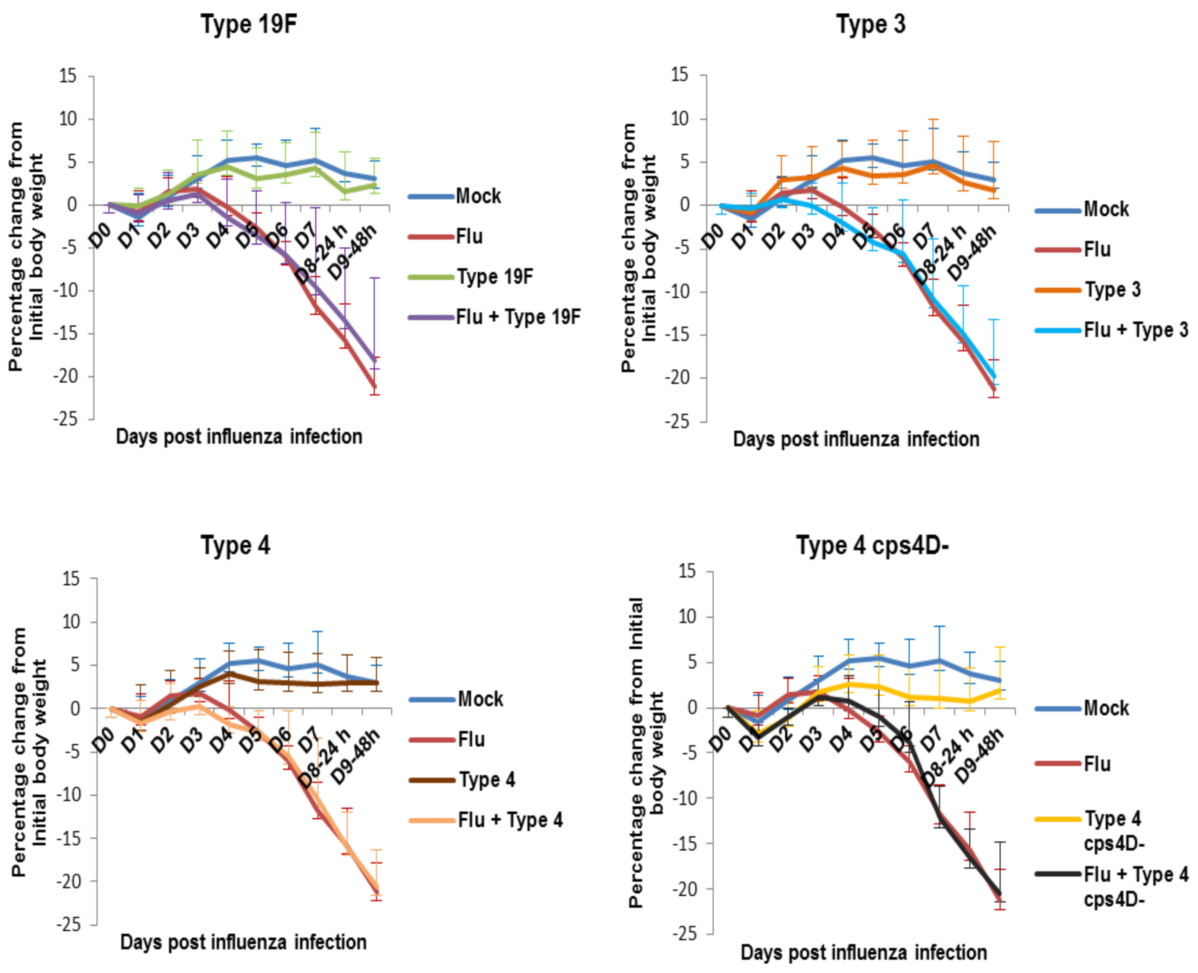

Supplementary Figure 2: Secondary infections with four strains of *S. pneumoniae* lead to body weight loss of mice similar to primary influenza infection. Mice were subjected to secondary infections with the four pneumococcal strains following primary influenza infection. All the influenza-infected mice suffered similar body weight loss, irrespective of the presence or absence of secondary pneumococcal infection. Values indicate means ± SE (*n* = 6 per group).

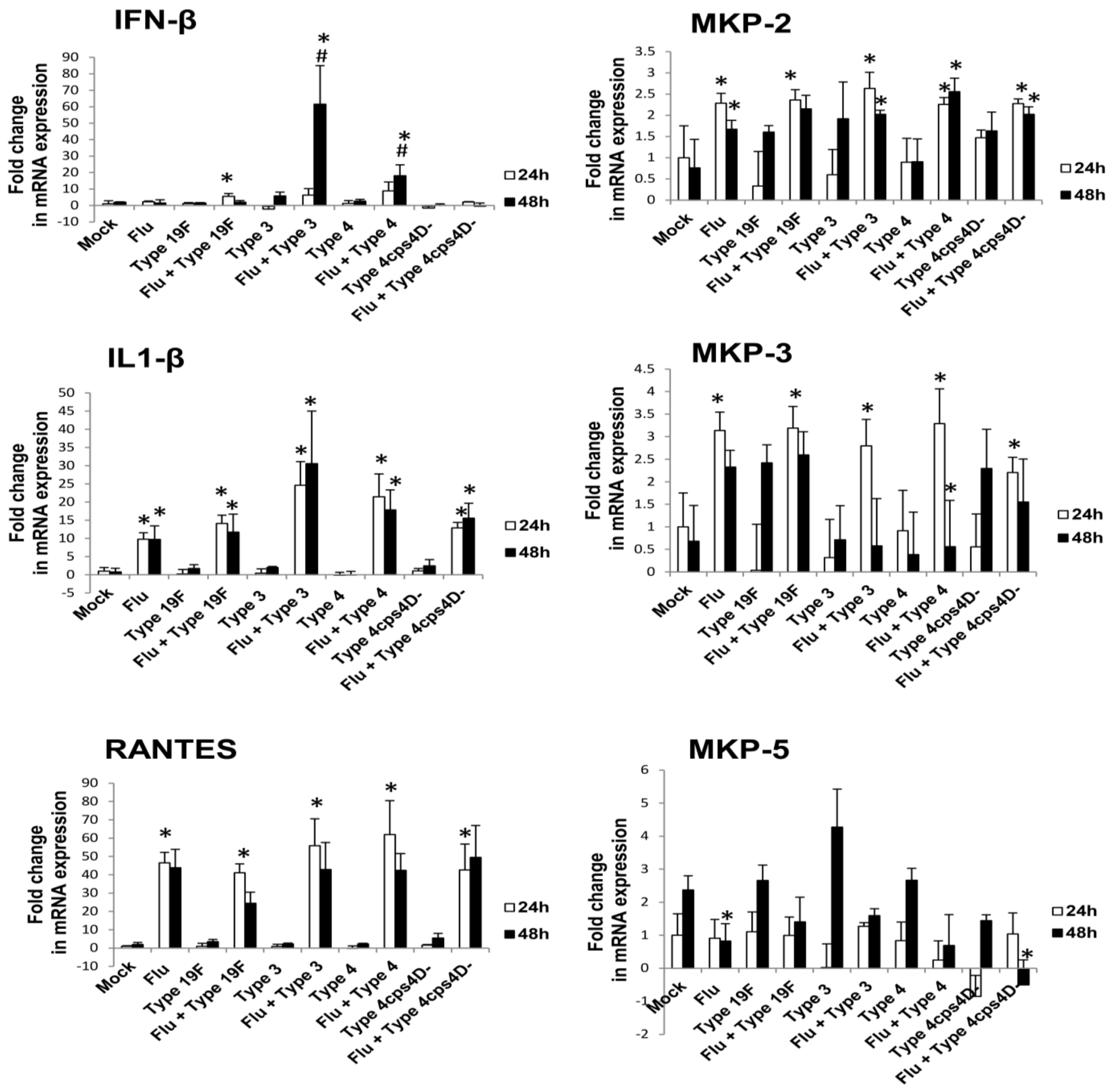

Supplementary Figure 3: Secondary infections with virulent pneumococci modulate gene expression of cytokines and cytokine-regulatory factors. Profiles of mRNA expression of pro-inflammatory cytokines and cytokine-regulatory MAP kinase phosphatases (MKP) were generally upregulated, especially in secondary infections. Interestingly, MKP-3 was downregulated after secondary infection with serotypes 3 and 4 at 48 hours, suggesting dysregulation of inflammatory cytokines. Values indicate means ± SE (*n* = 4 per group). *P* value < 0.05, * indicates significance over mock control, # denotes significance over secondary infection with serotype 19F.

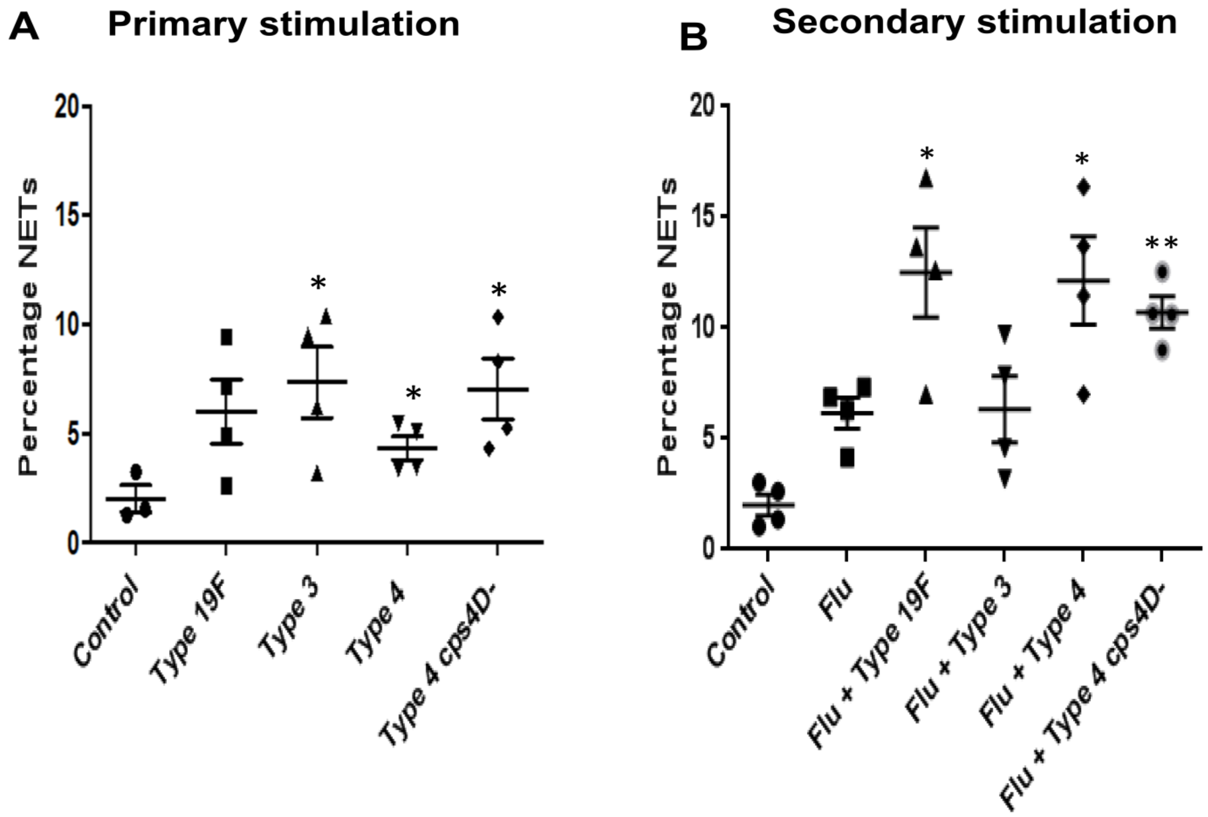

Supplementary Figure 4: NETs generated during primary and secondary stimulation by pneumococci *in vitro* show patterns different from *in vivo* observations. Bone marrow-derived neutrophils were stimulated *in vitro* to induce NETs. **A.** Primary stimulation of neutrophils with four pneumococcal strains was performed each at MOI of 1. Serotype 3 induced the highest NETs, while 4cps4D- induced more NETs than its wild-type. **B.** NETs were induced using an *in vitro* model of primary influenza stimulation followed by secondary pneumococcal infection. Bone marrow-derived neutrophils were stimulated for 2 hours using BALF from influenza-infected mice (day 5) to induce NETs. *S. pneumoniae* were then added at MOI of 1, and incubated for 2 h. Secondary infection with serotypes 19F, 4 and 4cps4D-induced significantly greater NETs compared to stimulation with influenza virus alone. Values indicate means ± SE (n = 3 per group). * $P < 0.05$, ** $P < 0.01$.

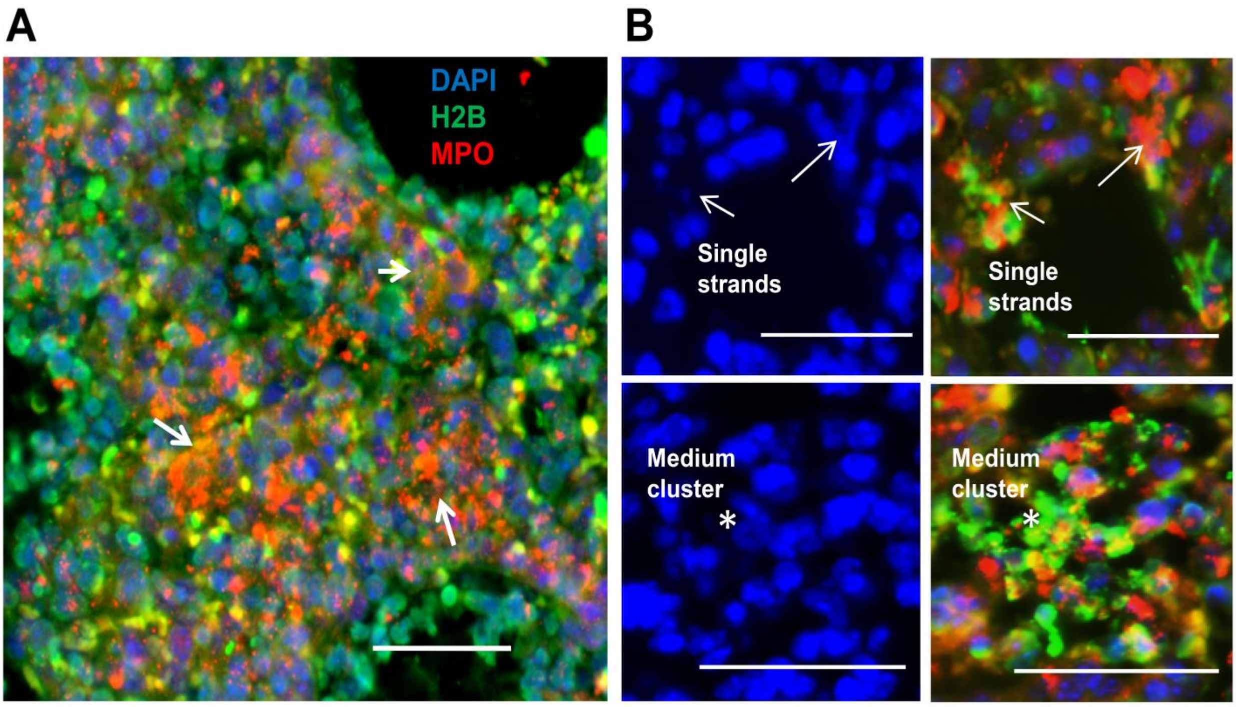

Supplementary Figure 5: Representative images of NETs used for scoring. A. Arrows indicate NETs in the lung sections after triple immunolabeling for histone H2B (H2B = green), myeloperoxidase (MPO = red), and nucleic acid (DAPI = blue). **B.** Representative images to demonstrate single strands (arrows) and clusters (asterisks). Scale bars = 50 µm. See NETs scoring criteria in Supplementary Table 1.

Supplementary Table 1: Scoring criteria for semi-quantitative analyses of NETs in lung sections.

Category	Number	Area (μm^2)	Score
None	N.A.	N.A.	0
Single strand	<5	N.A.	1
Single strand	≥5	N.A.	2
Cluster, small	1	<500	2
Cluster, small + single strands/clusters	>1	<500	4
Cluster, medium	1	500-5000	6
Cluster, medium + small cluster/strands	>1	500-5000	8
Cluster, large ± small cluster/strands	N.A.	>5000	10

Total NETs score = Sum of 20 fields per sample. N.A. = not applicable.

Supplementary Table 2: List of primers used in real-time PCR analyses.

Gene	Primers	Sequence (5′ – 3′)
IFN-β	Forward	CCCTATGGAGATGACGGAGA
	Reverse	CTGTCTGCTGGTGGAGTTCA
IL-1β	Forward	CAACCAACAAGTGATATTCTCCATG
	Reverse	ATCCACACTCTCCAGCTGCA
CCL-5 [RANTES]	Forward	ATATGGCTCGGACACCA
	Reverse	ACACACTTGGCGGTTCCT
MKP-2	Forward	TCTAAAACCAAGGCCCTGGC
	Reverse	GCCTCCTCCAGCCTCACCCG
MKP-3	Forward	GAGCTGGGCAACGAACGGCT
	Reverse	ACCGGGAAGGAAGGCTGGCT
MKP-5	Forward	GCTGTCCACATTAACTGTGCCG
	Reverse	TGGGCGTTAGCTCTGCGTTCTC
GAPDH	Forward	GACAACTTTGGCATTGTGG
	Reverse	ATGCAGGGATGATGTTCTG

SUPPLEMENTARY EXPERIMENTAL PROCEDURES

India ink staining to visualize capsule

Mid-logarithmic phase *S. pneumoniae* serotypes 3, 4 and 19F were mixed with a drop of India ink stain, and visualized immediately under 1000× magnification using a bright-field microscope. Capsules were seen as bright halo surrounding the bacterial cell in contrast to the dark background.

PCR amplification of *cps4D* gene

Five colonies of wild-type and cps4D- mutant of serotype 4 of *S. pneumoniae* were re-suspended with 50 µl nuclease-free water, and incubated at 95°C for 5 min to obtain DNA. The lysate (2 µl) was mixed with 23 µl of PCR master mix comprising 1× GoTaq green buffer in H_2O, 10 mM dNTP, 1.25 U GoTaq polymerase, 10 µM primers, and 25 mM $MgCl_2$ (Promega). The mixture was subjected to PCR amplification: denaturation at 95°C for 1 min, followed by 35 cycles each at 95°C for 20 sec, 55°C for 20 sec, 72°C for 20 sec, and a final extension at 72°C for 10 min. Specific forward primer (5′-ATAACCGGACCTTCTGAATC-3′) and reverse primer (5′-GAATATACGAGTACCACGCGA-3′) were used. The amplicons were subjected to 1.2% agarose gel electrophoresis to visualize the specific target fragment (1728 bp). Absence of this amplification product was considered as confirmation of gene deletion.

Isolation of bone marrow-derived neutrophils and induction of NETs

Briefly, single cell suspensions of bone marrow of donor mice were collected in Dulbecco's PBS without Ca^{2+}/Mg^{2+} (Biowest) after homogenizing using a 22G needle. The cell suspension was then carefully layered on discontinuous Percoll gradient (78%, 69% and 52%

v/v Percoll), and centrifuged at 1,500 *g* for 30 min. Mature neutrophils were recovered from the interphase between 69% and 78% Percoll. The purity of mature neutrophils was more than 85% as assessed by modified Giemsa staining. The cells were then fixed with 4% paraformaldehyde, washed, permeabilized with 0.5% Triton X-100, and stained with 1:500 dilutions of primary antibodies against MPO (rabbit polyclonal, Abcam) and histone H2B (mouse monoclonal, Abcam) at room temperature for 1 h. After 3 washes with 1× TBS, the cells were incubated with 1:250 dilutions of anti-rabbit Alexa Fluor 555 and anti-mouse Alexa Fluor 488 (Molecular Probes) along with DAPI at room temperature for 1 h. The slides were then mounted with anti-fade mounting medium (Invitrogen), and examined under a fluorescent microscope at 4000× magnification. NETs were enumerated in at least 10 fields per sample, and represented as percentage of total neutrophils.

Quantification of NETS in lung sections

NETs were stained in lung sections as described previously [14]. Lungs sections were deparaffinized, permeabilized with 0.025% Triton X-100, and stained with 1:250 dilutions of antibodies against histone H2B and MPO at 4°C overnight. Secondary staining was performed using Alexa Fluor 488 and Alexa Fluor 555 (1:250) with DAPI at room temperature for 1 hour. The sections were mounted with anti-fade mounting medium, and scanned using a high-resolution MIRAX MIDI system (Carl Zeiss). At least 20 fields from the whole section were captured using a Pannoramic Viewer, and NETs were scored using predetermined criteria based on their morphologic appearance as individual strands or clusters (Supplementary Figure 5 and Table 1). ImageJ was employed to measure the area of clusters. The total score was calculated as the sum of scores of 20 fields.
